# Successful treatment with bortezomib for POEMS syndrome, overcoming complicated severe heart block

**DOI:** 10.1002/ccr3.7004

**Published:** 2023-03-02

**Authors:** Yuichi Nakamura, Yoshihiro Itoh, Naoki Wakimoto, Ryu Kanno, Shinichirou Iida, Keiji Yamamoto

**Affiliations:** ^1^ Department of Hematology Saitama Medical University Hospital Moroyama Japan; ^2^ Department of Cardiovascular Medicine Saitama Medical University Hospital Moroyama Japan

**Keywords:** AV block, bortezomib, heart block, POEMS syndrome

## Abstract

Cardiac bradyarrhythmia and conduction disorder may be rare, but recurrent adverse events caused by bortezomib. Here we report a case with POEMS syndrome presenting severe heart block after bortezomib plus dexamethasone therapy. After permanent pacemaker implantation, bortezomib was restarted and maintained, resulting in sustained complete response for POEMS syndrome.

## Graphical abstract

Cardiac bradyarrhythmia and conduction disorder may be rare, but recurrent adverse events in bortezomib‐containing therapy for hematological diseases. Physicians should be aware of it to give appropriate treatment.

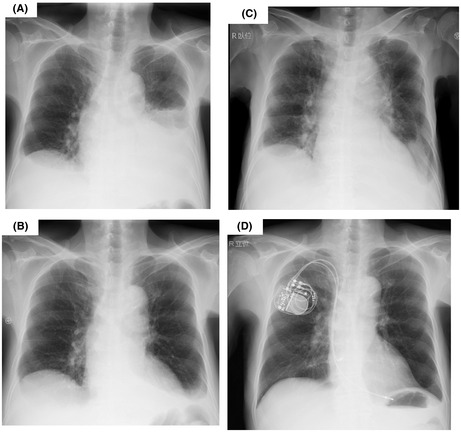

## 1 INTRODUCTION

POEMS syndrome is a rare plasma cell dyscrasia characterized by polyneuropathy, organomegaly, endocrinopathy, monoclonal gammopathy, and skin changes.^1^, ^2^ The pathophysiology of the syndrome is not well understood, but the elevation of vascular endothelial growth factor (VEGF) appears to be an important role in pathogenesis.^1^, ^2^, ^3^ Extravascular volume overload, including ascites, pleural effusion, and peripheral edema is observed in 29–87% of patients and often leads to severe morbidity.^1^, ^2^

Patients with POEMS syndrome in advanced disease need therapy to eradicate underlying clonal plasma cells for disease control. Systemic anti‐plasma cell therapies include high‐dose chemotherapy with autologous stem cell transplantation, alkylators, immunomodulatory drugs, or bortezomib.^1^, ^2^ Although standard front‐line treatment has not established, remarkable responses with bortezomib‐based therapy were shown recently.^4^, ^5^, ^6^, ^7^

Bortezomib is a proteasome inhibitor used as one of the key drugs in treatments for plasma cell dyscrasia such as multiple myeloma and the common adverse effects are peripheral neuropathies, asthenic conditions, and gastrointestinal disturbances. Cardiac adverse events of bradyarrhythmia and conduction disturbance with bortezomib were rarely reported.^8^, ^9^, ^10^, ^11^, ^12^, ^13^, ^14^ Here we present a case with POEMS syndrome who developed severe heart block with bortezomib plus dexamethasone (BDex) therapy. After permanent pacemaker implantation, BDex was restarted and maintained, resulting in sustained complete response.

## 2 CASE PRESENTATION

A 75‐year‐old female was admitted to our hospital with the chief complaints of abdominal distention and leg edema for 3 months. She had also been aware of dysesthesia in the limbs over a year. She had history of hypertension, hyperlipidemia, and left mammalian cancer which was treated with surgical resection at the age of 48. At the age of 74, she was affected with anterior‐septal myocardial infarction, which was treated with coronary artery angioplasty. Since then, she had been receiving anti‐platelet therapy with aspirin and clopidgrel. On physical examination, she presented cervical and axillary lymph nodes swelling (the largest measuring 3 cm in long axis), mild scleroderma in face to neck, massive ascites, and prominent edema in lower extremities.

**FIGURE 1:**
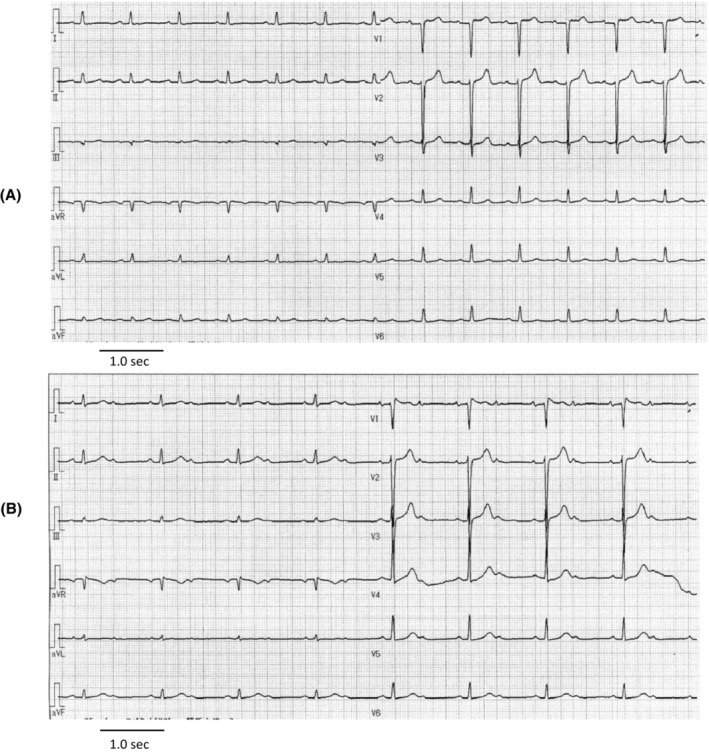
(A) 12‐lead electrocardiogram of the patient at the diagnosis of POEMS syndrome. Poor R progression with normal sinus rhythm (B) 12‐lead electrocardiogram of the patient on Day 15 in the first cycle of BDex therapy. 2:1 atrioventricular block with a ventricular rate of 49 bpm.

**FIGURE 2:**
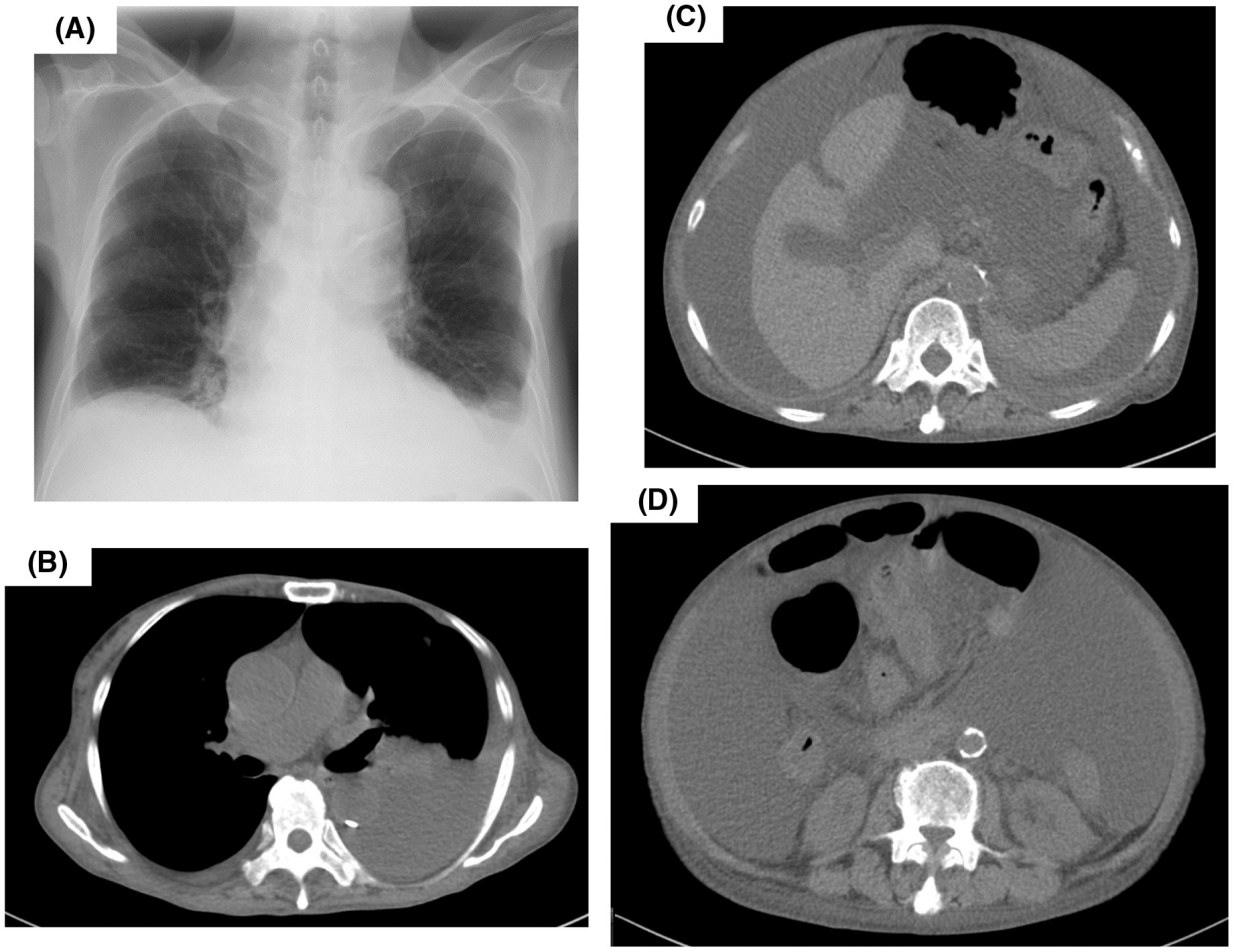
(A) Chest X‐ray (B) Chest CT on admission, exhibiting left pleural effusion. (C and D) Abdominal CT on admission, exhibiting massive ascites.

Peripheral blood showed hemoglobin level 10.9 g/dL, white blood cells 5.4 × 10^9^/L, and platelets 191 × 10^9^/L. Serum level of albumin was 3.4 g/dL and estimated glomerular filtration rate (eGFR) was decreased to 24.3 mL/min/1.73m^2^. Liver function tests were normal. Thyroid examination showed decrease of serum free T3 (1.54 pg/mL; normal range 2.15–4.24 pg/mL) and free T4 (0.89 ng/dL; normal range 1.00–1.70 ng/dL) and elevation of TSH (10.43 μIU/mL; normal range 0.39–3.98 μIU/mL). Plasma brain natriuretic peptide (BNP) level was slightly elevated to 53.0 pg/mL (normal range < 18.4 pg/mL). Electrocardiogram presented normal sinus rhythm with heart rate of 80 beats per minute (bpm) and poor R wave progression in V2 and V3 leads (Figure 1A). Left ventricular systolic function was normal with an ejection fraction of 78% and no signs indicating cardiac amyloidosis or other infiltrative diseases on echocardiogram. Chest x‐ray and computed tomography (CT) exhibited left pleural effusion (Figure 2A,B). Abdominal CT presented massive ascites pooling (Figure 2C,D).

Although levels of serum immunoglobulin were normal (IgG 1186 mg/dL, IgA 330 mg/dL, IgM 83 mg/dL), IgA‐λ monoclonal protein was detected on serum immunofixation. Bone marrow aspiration revealed slight increase of plasma cells (2.0%). An enlarged cervical lymph node was biopsied and subjected to pathological examination, which showed increase of lymphoid follicles in number, onionskin‐like structure in germinal centers, and vascular proliferation in interfollicular areas with sclerotic blood vessels penetrating into follicles. These findings coincided with those in hyaline‐vascular type of Castleman disease. Serum level of VEGF was highly elevated to 2150 pg/mL. Nerve conduction study revealed that motor conduction velocity was slowed and motor neuron latency was prolonged in median, ulnar, and tibial nerves, indicating demyelinating peripheral polyneuropathy.

**FIGURE 3:**
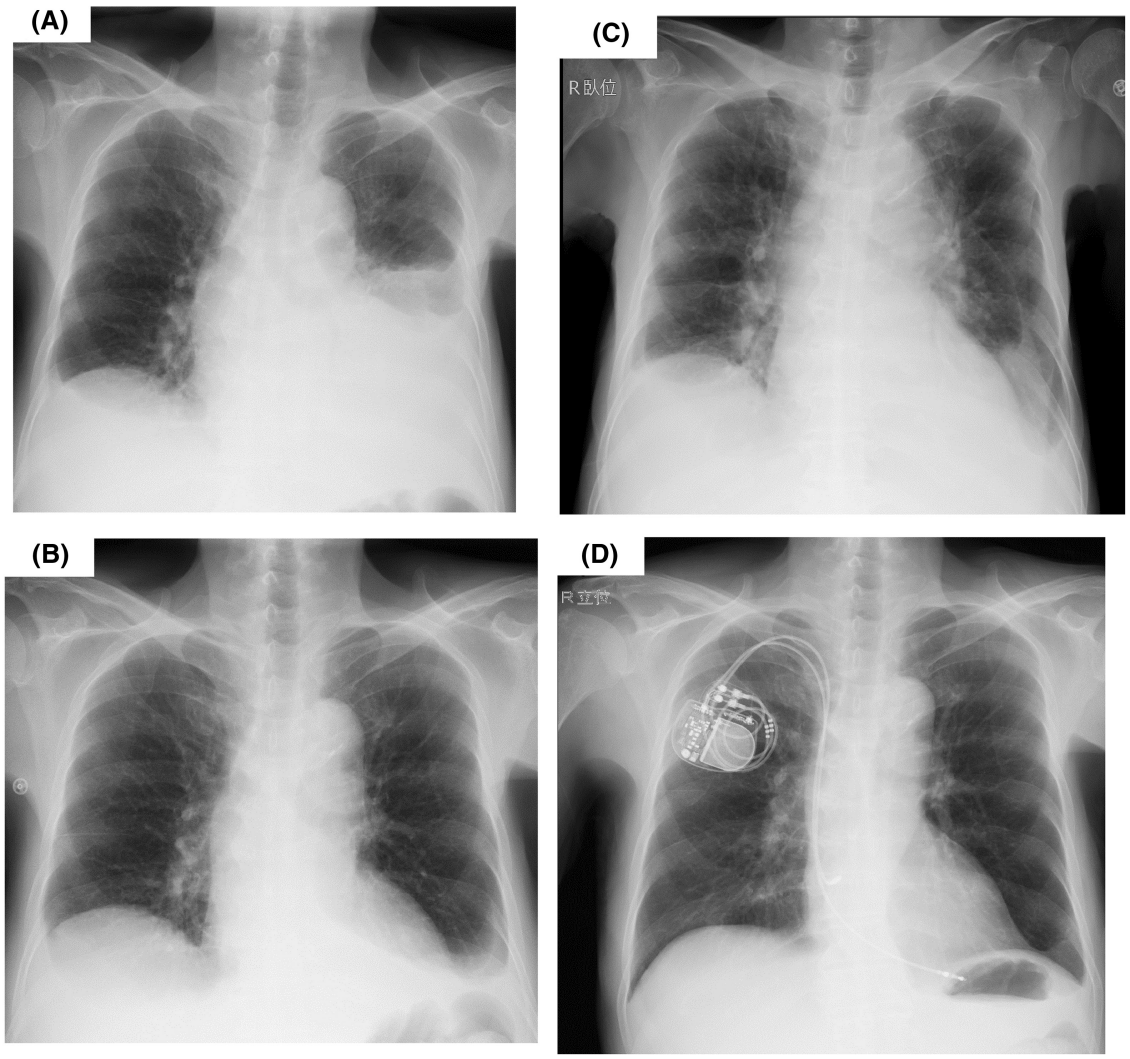
Chest x‐ray (A) At the start of BDex therapy, showing increase of left pleural effusion (B) On Day 14 of BDex, showing decrease of left pleural effusion. (C) On Day 18 of BDex (3 days after the onset of AV block), showing cardiac enlargement and the recurrence of pleural effusion. (D) After the permanent pacemaker implantation, showing the disappearance of cardiac enlargement, and pleural effusion.

The patient was diagnosed with POEMS syndrome, fulfilling of the mandatory criteria of peripheral polyneuropathy and monoclonal plasma cell proliferation producing λ‐type light chain, with major criteria of Castleman disease and VEGF elevation and minor criteria of extravascular volume overload (peripheral edema, pleural effusion, and ascites), endocrinopathy (hypothyroidism), and skin change (scleroderma). She received oral medication of levothyroxine for hypothyroidism and diuretics (spironolactone and furosemide) for fluid retention. However, she developed dyspnea with rapid increase of ascites and left pleural effusion (Figure 3A). To eradicate clonal plasma cells, bortezomib plus dexamethasone (BDex) therapy was started. Bortezomib was given intravenously at a reduce dosage of 1.0 mg/m^2^ on Days 1, 4, 8, and 11, in combination with oral dexamethasone 20 mg on Days 1, 2, 4, 5, 8, 9, 11, and 12, of a 21‐day cycle.

After the start of BDex therapy, the patient had relieved from dyspnea with rapid reduction of edema, ascites, and pleural effusion (Figure 3B). Lymphadenopathy was also disappeared quickly. However, on Day 15 of BDex, when cumulative dose of bortezomib was 4.0 mg/m^2^, she developed bradycardia and electrocardiogram revealed 2:1 atrioventricular (AV) block with a ventricular rate of 41–49 bpm (Figure 1B). On Day 18, chest X‐ray presented cardiac enlargement and the recurrence of pleural effusion (Figure 3C). Plasma BNP level elevated to 397 pg/mL, but repeated examinations showed normal ventricular systolic function without any defective wall motion on echocardiogram and no elevation of serum creatine kinase (CK)‐MB level.

BDex therapy was once discontinued and the severe heart block was treated with temporary transcutaneous pacing and subsequent permanent pacemaker implantation. She had recovered from bradycardia and compensatory cardiac volume overload was canceled with decrease of plasma BNP level (Figure 3D). Nineteen days after the pacemaker implantation, BDex therapy was restarted. After the second cycle of BDex therapy, monoclonal protein was not detected on serum immunofixation and serum level VEGF was decreased to 454 pg/mL. She had got free from any symptoms and levothyroxine and diuretics was stopped. She was discharged after three cycles of BDex and the therapy has been continued for 9 years with prolongation of interval of bortezomib administration to 2 months until now. Signs of relapse has not been observed with serum VEGF level of 247–272 pg/mL.

On follow‐up electrocardiogram examinations, the patient always presented pacemaker rhythm without restoration of her spontaneous rhythm, indicating that the injury of impulse conduction system had got irreversible.

## 3 DISCUSSION

POEMS syndrome is a multisystem disorder due to an underlying plasma cell dysplasia. The pathophysiology of the syndrome is not well understood, but VEGF appears to be an important role in pathogenesis.^1^, ^2^, ^3^ Disorders of VEGF, inducing a rapid and reversible increase in vascular permeability, may lead to edema, ascites, and pleural effusion.

Systemic anti‐plasma cell therapies including high‐dose chemotherapy with autologous stem cell transplantation, alkylators, immunomodulatory drugs, and bortezomib have been shown to be effective for POEMS syndrome, but standard front‐line treatment has not established.^1^, ^2^ Recently utility of bortezomib‐containing regimens had been reported.^4^, ^5^, ^6^, ^7^ Bortezomib is a proteasome inhibitor used as one of key drugs in treatments for plasma cell dyscrasia such as multiple myeloma. Besides the anti‐tumor cell activity, bortezomib was shown to downregulate *VEGF* gene expression and inhibit VEGF secretion.^8^ In the largest series consisted of 69 patients with POEMS syndrome, front‐line therapy with BDex was shown to be highly effective with 46.4% of hematological complete response and a good safety profile.^7^ The study also presented that pleural effusion, pulmonary hypertension, and impaired renal function recovered quickly, contributing to the promising survival for high‐risk patients.

In the present case with POEMS syndrome, BDex was chosen as the front‐line therapy for the progressive disease with extravascular volume overload. Immunomodulatory drugs (lenalidomide or thalidomide) were not adapted considering thrombotic risk with use the drugs in addition to the history of myocardial infarction. BDex therapy resulted in the rapid reduction of fluid retention and the improvement of general condition, but after the start of the therapy, she developed severe AV block with congestive heart failure due to bradycardia. The exact cause of AV block in this case was not identified. Newly occurring myocardial infarction or myocarditis was unlikely as she showed normal ventricular function on echocardiogram and no elevation of CK‐MB. Considering the clinical time course, therapy‐related, especially bortezomib‐induced, cardiac injury was thought to be most probable.

The common adverse effects with bortezomib are peripheral neuropathies, asthenic conditions, and gastrointestinal disturbances. Cardiac complications with bortezomib were also recurrently reported,^9^, ^10^ although the frequencies differed among the studies. Orciuolo et al. reported that 8 (11.6%) of the 69 patients with hematological disease treated with bortezomib developed serious cardiac side effects including heart failure, angina, atrial fibrillation, and bradycardia.^9^ Proteasome inhibitors are thought to cause cardiotoxicity through the unfolded protein response, leading to apoptosis in cardiac myocytes. Heart block or bradyarrhythmia with the use of bortezomib was rare. To our best knowledge, only six cases have been reported in the literature (Table 1).^9^, ^11^, ^12^, ^13^, ^14^ The mechanisms causing conduction disturbance with drug administration remains unknown. In the present case, the cardiac injury seems to be restricted to impulse conduction system, as echocardiogram showed normal ventricular function throughout the clinical course. Scarred regions by old myocardial infarction might have induced susceptibility for conduction disturbance.

**TABLE 1:** Previous and current cases presenting AV block and/or serious bradycardia

Case	Reference	Age (years)	Gender	Disease	No. of cycles of bortezomib containing regimen	Diagnosis of cardiac complication
1	9	73	F	MM	5	AV block
2	9	66	M	NHL	4	Bradycardia
3	12	87	F	MM	10	Complete AV block
4	13	65	F	MM	2	Complete AV block
5	14	66	M	MM	6	Complete AV block
6	Present case	75	F	POEMS syndrome	1	2:1 AV block

*Abbreviations*: MM; multiple myeloma, NHL; non‐Hodgkin lymphoma.

*Note*: An additional case was reported, but clinical information was not available.^11^

Bradyarrhythmia in POEMS syndrome before anti‐plasma cell treatment was also reported in two cases.^15^, ^16^ One case with pleural and pericardial effusion also presented left ventricular dysfunction with increased T1 values and elevated cardiac extracellular volume on cardiac magnetic resonance examination, suggesting myocardial edema caused by POEMS syndrome.^16^ In our case, such mechanism was unlikely as the onset of AV block was during the course of the disease improvement.

In the present case, after the improvement of bradyarrhythmia and heart failure with permanent pacemaker implantation, we decided to restart the bortezomib‐based therapy with careful monitoring of cardiac function, as it was postulated that further cardiac conduction disturbance would be overcome with implanted pacemaker and failure in POEMS syndrome with another therapy would lead to fatal clinical outcome. Continuation of the effective BDex therapy resulted in the sustained complete response with good general condition for long‐term over 9 years.

Cardiac bradyarrhythmia and conduction disorder may be rare, but recurrent adverse events in bortezomib‐containing therapy for hematological disease. Physicians should be aware of it to give appropriate treatment.

## 4 CONCLUSION

Here we present a case with POEMS syndrome who developed serious heart block with bortezomib plus dexamethasone (BDex) therapy. The patient with history of myocardial infarction was diagnosed with POEMS syndrome showing peripheral edema, pleural effusion, and massive ascites. With the start of BDex, fluid retention was quickly reduced, but on Day 15 of the therapy, she developed severe AV block which was treated successfully with temporary pacing and subsequent permanent pacemaker implantation. After that, bortezomib‐based therapy was restarted and continued, resulting in sustained complete response.

Cardiac bradyarrhythmia and conduction disorder may be rare, but recurrent adverse events in bortezomib‐containing therapy. Physicians should be aware of it to give appropriate treatment.

## AUTHOR CONTRIBUTIONS

**Yoshihiro Itoh:** Investigation. **Naoki Wakimoto:** Investigation. **Ryu Kanno:** Investigation. **Shinichirou Iida:** Investigation. **Keiji Yamamoto:** Investigation.

## CONFLICT OF INTEREST STATEMENT

The authors declare no conflicts of interest associated with this manuscript.

## ETHICS STATEMENT

Ethics approval is not applicable to this article as an observational study in which individuals are not identified.

## INFORMED CONSENT

Written informed consent was obtained from the patient for publication.

## PERMISSION TO REPRODUCE MATERIAL FROM OTHER SOURCES

Reproduction is not permitted.

## Data Availability

Data sharing is not applicable to this article as no new data were created or analyzed in this study.
